# Supplementary material for: KiSS-1 Modulation by Epigenetic Agents Improves the Cisplatin Sensitivity of Lung Cancer Cells
Source: Int J Mol Sci. 2024 May 6;25(9):5048. doi: 10.3390/ijms25095048 (PMC11084743; doi:10.3390/ijms25095048)
Supplement: Supplementary file 1 [file ijms-25-05048-s001.zip › ijms-2980356-supplementary.pdf]

Supplementary material

KiSS-1 modulation by epigenetic agents improves cisplatin sensitivity of lung cancer cells

Giovanni Luca Beretta[1,*], Desirè Alampi[1] , Cristina Corno[1], Nives Carenini[1], Elisabetta Corna[1] and Paola Perego[1,*]

[1] Molecular Pharmacology Unit, Department of Experimental Oncology, Fondazione IRCCS Istituto Nazionale dei Tumori, 20133 Milan, Italy; giovanni.beretta@istitutotumori.mi.it, dmalampi@gmail.com, cristina.corno@istitutotumori.mi.it, nives.carenini@istitutotumori.mi.it, elisabetta.corna@istitutotumori.mi.it, paola.perego@istitutotumori.mi.it

* Correspondence: paola.perego@istitutotumori.mi.it (PP); giovanni.beretta@istitutotumori.mi.it (GLB)

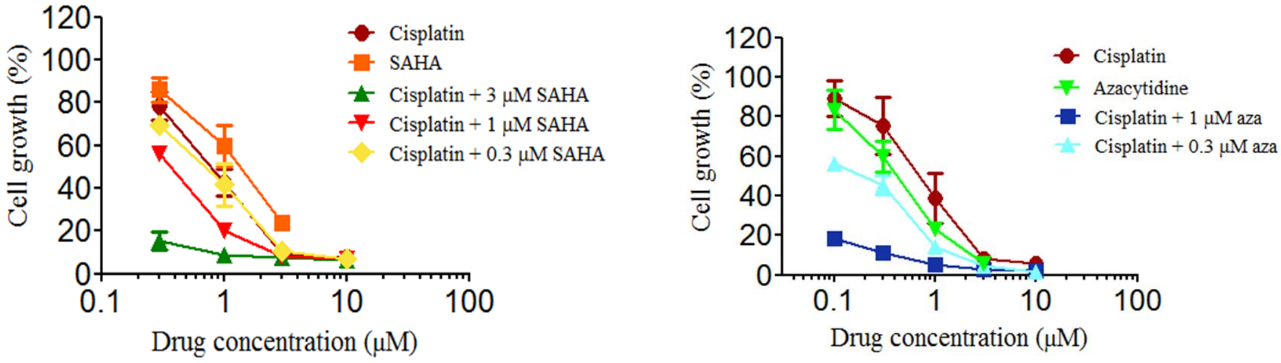

Figure S1. Cell sensitivity of H460 cells in the simultaneous treatment schedule with SAHA or AZA. Cell sensitivity was evaluated through cell growth inhibition assay. Cells were treated for 72 h with SAHA or AZA in simultaneous combination with cisplatin. After 72 h cells were counted with cell counter. The values reported in the graph represent the means of three experiments and the corresponding standard deviations.

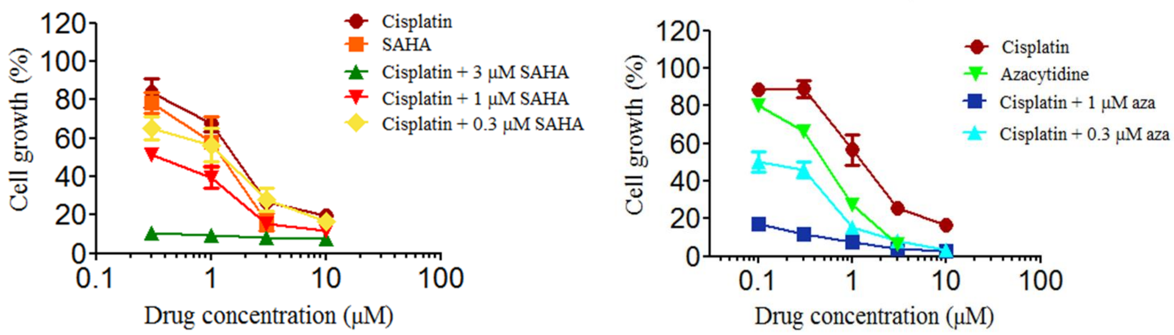

Figure S2. Cell sensitivity of H460 cells in the pre-incubation treatment schedule with SAHA and AZA. Cell sensitivity was evaluated through cell growth inhibition assay. Cells were treated for 24 h with SAHA or AZA, cells were co-incubated with cisplatin. After 48 h cells were counted with an automated cell counter. The values reported in the graph represent the means of three experiments and the corresponding standard deviations.

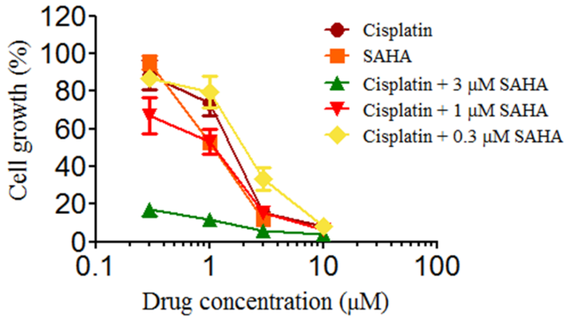

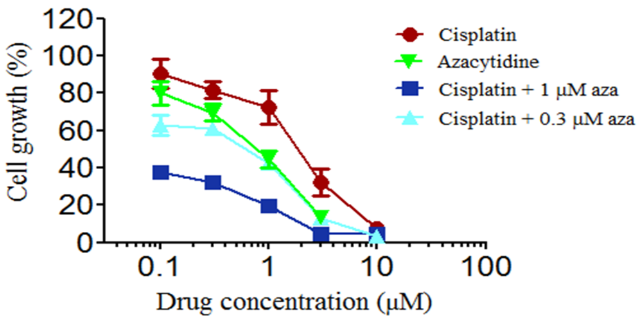

Figure S3. Cell sensitivity of H460/Pt cells in the simultaneous treatment schedule with SAHA and AZA. Cell sensitivity was evaluated through cell growth inhibition assay. Cells were treated for 72 h with SAHA or AZA in simultaneous combination with cisplatin. After 72 h cells were counted with an automated cell counter. The values reported in the graph represent the means of three experiments and the corresponding standard deviations.

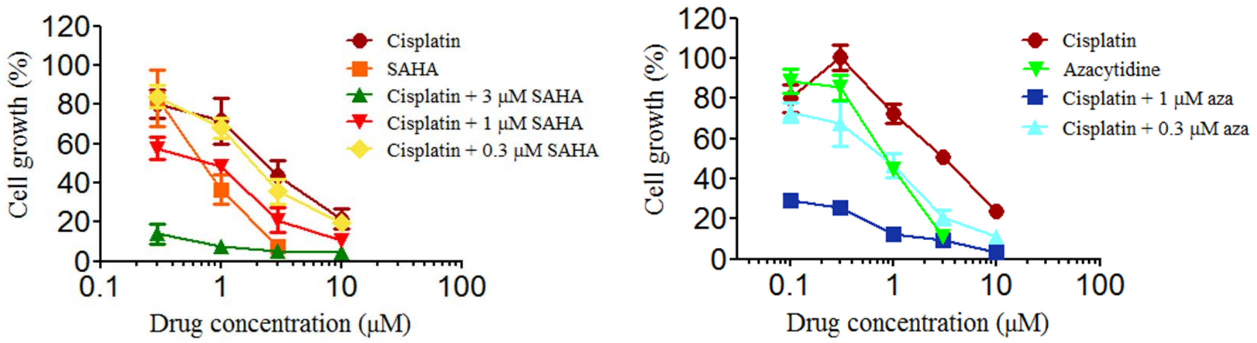

Figure S4. Cell sensitivity of H460/Pt cells in the pre-incubation treatment schedule with SAHA and AZA. Cell sensitivity was evaluated through cell growth inhibition assay. Cells were treated for 24 h with SAHA or AZA, cells were co-incubated with cisplatin. After 48 h cells were counted with an automated cell counter. The values reported in the graph represent the means of three experiments and the corresponding standard deviations.

Table S1. Heat map recapitulating the combination indexes reported in Table 2, 3, 4, 5 [a]

Table 2

**Cisplatin**	0.1 µM	0.3 µM	1 µM	3 µM	10 µM
**SAHA**					
0.3µM	-	1,32	1,15	0,67	1,44
1 µM	-	1,52	0,68	0,56	1,56
3 µM	-	0,71	0,58	0,82	1,46
**AZA**					
0.3 µM	0,98	1,02	0,49	0,44	0,51
1 µM	0,79	0,58	0,38	0,39	0,91

Table 3

**Cisplatin**	0.1 µM	0.3 µM	1 µM	3 µM	10 µM
**SAHA**					
0.3µM	-	0,98	1,44	0,81	1,06
1 µM	-	1,41	1,27	0,56	0,83
3 µM	-	0,65	0,63	0,63	0,82
**AZA**					
0.3 µM	0,83	0,83	0,3	0,26	0,22
1 µM	0,71	0,49	0,36	0,25	0,27

Table 4

**Cisplatin**	0.1 µM	0.3 µM	1 µM	3 µM	10 µM
**SAHA**					
0.3µM	-	1,42	2,34	1,42	1,33
1 µM	-	1,39	1,31	1,37	1,42
3 µM	-	1,45	1,24	1,07	1,39
**AZA**					
0.3 µM	1,11	1,48	0,97	0,46	0,42
1 µM	1,06	0,96	0,64	0,23	0,58

Table 5

**Cisplatin**	0.1 µM	0.3 µM	1 µM	3 µM	10 µM
**SAHA**					
0.3µM	-	4,56	2,18	1,01	1,15
1 µM	-	2,67	2,03	1,02	0,81
3 µM	-	1,79	1,16	0,92	0,94
**AZA**					
0.3 µM	1,06	1,62	0,64	0,3	0,27
1 µM	0,65	0,59	0,32	0,28	0,16

[a] CI lower than 0.85–0.90 indicate synergistic drug interactions (green). CI values higher than 1.20–1.45 indicate antagonism (red) CI between 0.91-1.19 reflect additive activity (orange). The methodology is described in Materials and Methods section.

Table S2. Heat map recapitulating the Western blot quantification of Figure 1[a]

H460 (Figure 1A)			
#	p53	Bax	Bcl2
Control	1	1	1
3µM SAHA	58,5328814	6,372439185	3,715862454
1µM SAHA	2,61841805	5,048594113	1,447191908
0.3µM SAHA	13,9062163	2,1596332	1,95808765
3µM Cisplatin	40,0134762	2,921091073	0,933653424
3µM SAHA+3µM Cisplatin	15,6661694	6,999680074	0,97187535
1µM SAHA+3µM Cisplatin	51,7923945	5,692568851	2,004082825
0.3µM SAHA+3µM Cisplatin	36,3119577	2,504923191	2,864747257

H460 (Figure 1A)			
#	p53	Bax	Bcl2
Control	1	1	1
1µM AZA	2,37857272	1,82600992	1,386508977
0.3µM AZA	1,94897223	1,0384375	1,294442138
0.1µM AZA	0,3946025	1,00001171	0,809567944
3µM Cisplatin	3,07964428	10,0176381	3,06064735
1µM AZA+3µM Cisplatin	4,60095277	8,19930144	1,29999986
0.3µM AZA+3µM Cisplatin	4,66567565	7,14074466	1,537262006
0.1µM AZA+3µM Cisplatin	2,8277553	9,02964459	1,255867925

H460/Pt (Figure 1B)			
#	p53	Bax	Bcl2
Control	1	1	1
3µM SAHA	9,11749713	0,77156442	1,89947515
1µM SAHA	5,10360426	1,3219479	3,04993431
0.3µM SAHA	5,36244705	1,92642309	1,74857312
10µM Cisplatin	49,494183	10,5052529	1,33415774
3µM SAHA+10µM Cisplatin	20,7621825	2,74383789	0,8802923
1µM SAHA+10µM Cisplatin	22,5218164	4,74848692	1,05606563
0.3µM SAHA+10µM Cisplatin	28,4356092	11,2671701	1,55092809

H460/Pt (Figure 1B)			
#	p53	Bax	Bcl2
Control	1	1	1
1µM AZA	1,5703189	1,0956618	0,9504123
0.3µM AZA	0,6172014	0,4337637	0,7497884
0.1µM AZA	2,6994968	0,7475805	1,3994632
10µM Cisplatin	11,277251	4,9517075	1,1457491
1µM AZA+10µM Cisplatin	16,544136	5,6671084	0,8990518
0.3µM AZA+10µM Cisplatin	16,053612	5,5261492	1,2097208
0.1µM AZA+10µM Cisplatin	16,342217	4,5159713	1,0960267

[a]The band intensity was quantified by using Image Studio Lite Ver.5.2 (LICOR Biosciences). Each band intensity of selected protein (e.g., p53, Bax, Bcl2) was divided to the band intensity of the corresponding actin. The values of treated samples were then normalized to the corresponding control untreated samples. The heat map has been generated using excel program (green>1 (increased expression); red<1 (reduced expression).
